# Prevalence of Firearm Ownership Among Individuals With Major Depressive Symptoms

**DOI:** 10.1001/jamanetworkopen.2022.3245

**Published:** 2022-03-21

**Authors:** Roy H. Perlis, Matthew D. Simonson, Jon Green, Jennifer Lin, Alauna Safarpour, Kristin Lunz Trujillo, Alexi Quintana, Hanyu Chwe, John Della Volpe, Katherine Ognyanova, Mauricio Santillana, James Druckman, David Lazer, Matthew A. Baum

**Affiliations:** 1 Department of Psychiatry and Center for Quantitative Health, Massachusetts General Hospital, Boston; 2 Department of Psychiatry, Harvard Medical School, Boston, Massachusetts; 3 Network Science Institute and Institute for Qualitative Social Science, Northeastern University, Boston, Massachusetts; 4 Department of Political Science, University of Pennsylvania, Philadelphia; 5 Department of Political Science, Northwestern University, Evanston, Illinois; 6 Harvard Kennedy School of Government, Cambridge, Massachusetts; 7 School of Communication and Information, Rutgers University, New Brunswick, New Jersey; 8 Department of Pediatrics, Harvard Medical School, Cambridge, Massachusetts; 9 Department of Epidemiology, T.H. Chan School of Public Health, Harvard University, Cambridge, Massachusetts; 10 Computational Health Informatics Program, Boston Children’s Hospital, Boston, Massachusetts

## Abstract

**Importance:**

Both major depression and firearm ownership are associated with an increased risk for death by suicide in the United States, but the extent of overlap among these major risk factors is not well characterized.

**Objective:**

To assess the prevalence of current and planned firearm ownership among individuals with depression.

**Design, Setting, and Participants:**

Cross-sectional survey study using data pooled from 2 waves of a 50-state nonprobability internet survey conducted between May and July 7, 2021. Internet survey respondents were 18 years of age or older and were sampled from all 50 US states and the District of Columbia.

**Main Outcomes and Measures:**

Self-reported firearm ownership; depressive symptoms as measured by the 9-item Patient Health Questionnaire.

**Results:**

Of 24 770 survey respondents (64.6% women and 35.4% men; 5.0% Asian, 10.8% Black, 7.5% Hispanic, and 74.0% White; mean [SD] age 45.8 [17.5]), 6929 (28.0%) reported moderate or greater depressive symptoms; this group had mean (SD) age of 38.18 (15.19) years, 4587 were female (66.2%), and 406 were Asian (5.9%), 725 were Black (10.5%), 652 were Hispanic (6.8%), and 4902 were White (70.7%). Of those with depression, 31.3% reported firearm ownership (n = 2167), of whom 35.9% (n = 777) reported purchasing a firearm within the past year. In regression models, the presence of moderate or greater depressive symptoms was not significantly associated with firearm ownership (adjusted odds ratio [OR], 1.07; 95% CI, 0.98-1.17) but was associated with greater likelihood of a first-time firearm purchase during the COVID-19 pandemic (adjusted OR, 1.77; 95% CI, 1.56-2.02) and greater likelihood of considering a future firearm purchase (adjusted OR, 1.53; 95% CI, 1.23-1.90).

**Conclusions and Relevance:**

In this study, current and planned firearm ownership was common among individuals with major depressive symptoms, suggesting a public health opportunity to address this conjunction of suicide risk factors.

## Key Points

**Question:**

How common is firearm ownership, a risk factor for suicide, among individuals with major depressive symptoms in the United States?

**Findings:**

In this cross-sectional survey study including 24 770 respondents, individuals with moderate depressive symptoms were as likely as those without to own firearms but more likely to have recently purchased a first firearm and to express interest in a purchase in the near future.

**Meaning:**

The findings of this study suggest that firearm ownership is common among individuals with depression in the United States, highlighting an opportunity to diminish suicide risk through focused interventions.

## Teaser

This cross-sectional survey study assesses the prevalence of firearm ownership among individuals with major depressive symptoms in the United States.

## Introduction

Firearm ownership has been recognized as a major risk factor for suicide attempt and suicide death for at least 3 decades.^1^ Rates of handgun ownership were strongly associated with suicide rates,^2^ and the trends in each of these rates were shown to be associated over time.^3^ Most notably, a study of California residents found hazard of suicide to be 3 times greater among men and 7 times greater among women if they owned a firearm.^4^ With the increase in firearm purchases observed during the COVID-19 pandemic,^5^ the potential effect of this risk factor has only increased.

Likewise, mood disorders, and depressive episodes in particular, are strongly associated with suicide risk.^6,7^ In some (but not all^2^) investigations, depression is also associated with gun ownership,^8^ consistent with another small study of young adults with a history of suicidality.^9^

Remarkably, little is known about the convergence of these 2 risk factors for suicide (ie, the extent of firearm ownership among individuals who are depressed). As a particularly high-risk group, if only because they have 2 major risk factors, gun owners with depression would seem to represent a prime opportunity for focused interventions to reduce risk. Such a focus may be critical given the challenges in developing reliable risk stratification and effective suicide prevention strategies.^10^ Indeed, a study^11^ of 135 US Army soldiers who died of suicide found that firearm ownership and modifiable aspects of ownership, such as gun storage, were associated with risk.^11^ To better understand the characteristics of individuals with depression who own or plan to purchase firearms, we used data from 2 waves of a large national survey conducted between April and July 2021. We sought to understand the prevalence of firearm ownership, including recent purchase (defined as purchase within the last 12-18 months) and plans for future purchase, among individuals with depressive symptoms and then to quantify the sociodemographic features associated with ownership in this group.

## Methods

### Study Design

Data were obtained from the COVID States Project,^12^ an academic consortium that has fielded a survey approximately every 4 to 6 weeks beginning in April 2020. Questions related to firearm ownership and purchasing were included in 2 waves, the first conducted between April 1 and May 3, 2021, and the second between June 9 and July 7, 2021. The survey was conducted online using a commercial vendor that aggregates panels, applying nonprobability sampling and representative quotas to approximate the distribution of age, sex, and race and ethnicity across each of the 50 states and the District of Columbia (for a comparison of nonprobability and probability sampling, see Kennedy and Caumont^13^; for studies demonstrating viability of nonprobability sampling with this design for similar investigations, see Coppock and McClellan^14^ and Berinsky et al^15^).

The institutional review board of Harvard University reviewed the study design and categorized it as exempt as a survey study with minimal risk to participants; survey participants provided their signed consent online prior to survey access. Reporting of results followed the American Association for Public Opinion Research (AAPOR) reporting guideline for survey studies. Of note, to minimize selection bias from participants who might have a particular interest (in firearms or mental health, for example), participants were not told about the survey topic before opting in to survey completion.

### Outcomes and Assessments

All survey participants completed the 9-item Patient Health Questionnaire (PHQ-9) as a validated measure of major depressive symptoms during the preceding 2 weeks.^16^ In primary care settings, a value of 10 or greater represents at least moderate depression with specificity of approximately 88% confirmed by a large meta-analysis from 2019^16,17^; this cutoff is often considered a threshold for treatment or referral and was applied in this study to maximize face validity given that it is designed as a screening, not a depression severity, instrument. Item 9 of the PHQ-9 asks about suicidal ideation, phrased as “thoughts that you would be better off dead, or thoughts of hurting yourself in some way”; for analysis, scores greater than 0 (ie, several days or more) were considered to represent the presence of suicidality.

The surveys included a yes or no question about current gun ownership (“Do you or a member of your household own a gun?”) and a gun purchase during the pandemic (“Did you or a member of your household buy a gun during the COVID-19 pandemic?”). Those with a gun purchase during the pandemic were asked to identify 1 or more reasons for the purchase from a list, including hunting, target shooting, protection against crime, protection against the government, concern about COVID-19, concern about lockdown and restrictions, concern about the US presidential election, and protection against someone they know personally. Respondents were also asked about the intention to purchase a gun in the near future (“How likely are you to purchase a gun in the next few months?”) with 4 responses: very likely, somewhat likely, somewhat unlikely, or very unlikely. For analysis, the somewhat likely or very likely category was compared with the somewhat unlikely or very unlikely category. To minimize survey length, participants were randomly assigned to questions such that not all respondents viewed all questions, while maintaining sufficient sample size to power primary analyses with 5 to 10 covariates even for subgroups.

Sociodemographic features, including race and ethnicity and sex, were identified by self-report that mapped to categories reflected in historical US census categories. Region (Northeast, South, Midwest, and West) and urban or rural status were assigned based on zip code using 2020 US census designations. Political ideology (from extremely liberal = 1 to extremely conservative = 7) was measured using a 7-point scale, with 4 representing “moderate, middle of the road” obtained from the American National Election Studies questions. Political party affiliation was determined by asking, “Generally speaking, do you think of yourself as a…” with Democrat, Republican, Independent, and other as options; for analytic purposes, other and independent were combined in a single category (independent or other).

### Survey Validation

To validate survey-based estimates of firearm ownership and recent firearm purchase, we compared our state-level estimates of each with 2 external criterion standard data sets, recognizing that available data on both firearm ownership and purchase in the United States are limited. For ownership, we used a 2020 RAND report estimating household firearm ownership rates by state through 2016.^21^ These estimates are based on a combination of probability-sampled polling data, rates of suicide, permit issuance, and background checks, in an effort to overcome the limitations of probability-sampled data alone. For firearm purchasing, we used state-level background check data from the US Federal Bureau of Investigation (FBI) obtained from the period between February 2020 and March 2021 (ie, prior to the early April 2021 survey wave that asked about ownership). Because the FBI does not release these data in electronic form, data were accessed from GitHub,^22^ which extracts numbers from the public PDFs. These data, albeit the most precise available data regarding purchases, distinguish handgun from long gun and multiple gun purchases; as such, it is not possible to know how many unique individuals purchased firearms, as distinct from multiple firearm purchases by a given individual. In addition, not all individuals purchasing firearms are subject to background check.^23^ Still, these data have been used in prior reports of state-level purchases.^24^ For the present effort, we assumed that even if they do not allow a precise estimate of household purchases, they should still represent a reasonable proxy for such purchases. Firearm purchases were divided by the total number of adults 18 years of age or older for each state based on 2018 US Census American Community Survey results.^18^ Of note, in a subset of states that has been challenging to survey using population-representative quotas (8 states plus the District of Columbia), a shorter form of the survey was used that did not include firearm questions. The results in all of the remaining states (reweighted to reflect state-level demographic features) were correlated with the public data on ownership and purchases.

### Statistical Analysis

Although a small number of survey participants (1387 of 24 770 [5.6%]) were present in both waves, for purposes of analysis, we included only the first response per participant. The primary outcome of interest was current gun ownership; we also examined the recent purchase of a gun by individuals who did not previously own one and the interest in purchasing a first gun among those who did not own one. We used logistic regression to estimate crude odds ratios (ORs) for the association between the presence of moderate or greater major depressive symptoms and each outcome, as well as ORs adjusted for sociodemographic features and political orientation. The specific sociodemographic features included in the regression models included age in years; sex; race and ethnicity (captured using US census categories); level of education; employment status; household income; urban, suburban, or rural location; and region. The characteristics of political orientation included in the regression models included party affiliation and ideology. These variables were included on the basis of prior survey waves examining COVID-19–related behaviors implicating sociodemographic features and political beliefs.^12^ We also examined features associated with the greater likelihood of each outcome among individuals with major depressive symptoms (ie, conditional on meeting criteria for moderate or greater depression, what were the features associated with gun ownership?). To test whether these features were differentially associated with gun ownership among individuals with depression, we repeated these models among all survey respondents, adding terms for interaction of individual features with the presence or absence of depression. In sensitivity analysis, we also considered effects solely among the subset of individuals who reported depressive symptoms as well as suicidality.

For all regression models, the survey results were reweighted using interlocking national weights for age, sex, and race and ethnicity, education, and region based on the 2019 US Census American Community Survey^18^ (Table 1), using the survey package in R, version 4.0 (The R Project for Statistical Computing),^19^ a standard approach shown to perform well for nonprobability samples.^20^ Statistical significance was defined as a nominal 2-sided *P* value of less than .05.

**Table 1.** Sociodemographic Features of Survey Participants With or Without Moderate or Greater Symptoms of Major Depressive Disorder

Characteristic	Participants, No. (%)			*P* value	US values, %^a^
	Less than moderate depression (n = 17 841)	Moderate or greater depressive symptoms (n = 6929)	Total (n = 24 770)		
Gun ownership	5668 (31.8)	2167 (31.3)	7835 (31.6)	.45	NA
Recent gun purchase^b^	1083 (6.1)	777 (11.2)	1860 (7.5)	<.001	NA
Planned gun purchase^c^	1495 (20.2)	1014 (30.6)	2509 (23.4)	<.001	NA
Age, mean (SD), y	48.8 (17.4)	38.2 (15.2)	45.8 (17.5)	<.001	NA^d^
Sex				<.001	
Female	11 410 (64.0)	4587 (66.2)	15 997 (64.6)	<.001	51.6
Male	6431 (36.0)	2342 (33.8)	8773 (35.4)		48.4
Race and ethnicity					
Asian	832 (4.7)	406 (5.9)	1238 (5.0)	<.001	6.3
Black	1957 (11.0)	725 (10.5)	2682 (10.8)		12.0
Hispanic	1213 (6.8)	652 (9.4)	1865 (7.5)		16.6
White	13 434 (75.3)	4902 (70.7)	18 336 (74.0)		62.9
Other	405 (2.3)	244 (3.5)	649 (2.6)		2.2
Education (some college)	8149 (45.7)	2549 (36.8)	10 698 (43.2)	<.001	58.0
Currently employed^e^	9943 (55.8)	4040 (58.3)	13 983 (56.5)	<.001	NA
Income, $10 000, mean (SD)	7.10 (5.07)	6.31 (5.34)	6.88 (5.16)	<.001	NA
Region					
Midwest	4499 (25.2)	1629 (23.5)	6128 (24.7)	<.001	21.0
Northeast	2960 (16.6)	1033 (14.9)	3993 (16.1)		18.0
South	6603 (37.0)	2694 (38.9)	9297 (37.5)		38.0
West	3779 (21.2)	1573 (22.7)	5352 (21.6)		24.0
Urbanicity					
Rural	2817 (15.8)	1064 (15.4)	3881 (15.7)	<.001	14.0
Suburban	10 481 (58.7)	3932 (56.7)	14 413 (58.2)		55.0
Urban	4543 (25.5)	1933 (27.9)	6476 (26.1)		31.0
Political ideology, mean (SD)^f^	4.01 (1.65)	3.48 (1.67)	3.87 (1.67)	<.001	
PHQ-9					
Suicidality	1260 (7.1)	4270 (61.6)	5530 (22.3)	<.001	NA
Suicidality score, mean (SD)	0.08 (0.30)	1.15 (1.10)	0.38 (0.80)	<.001	NA
Mean score, mean (SD)	3.18 (2.97)	15.98 (4.81)	6.76 (6.77)	<.001	NA

Abbreviations: NA, not applicable; PHQ-9, 9-item Patient Health Questionnaire.

^a^ National values obtained from 2018 US Census American Community Survey.

^b^ No data on new gun purchase for 4 respondents (2 with depressive symptoms).

^c^ No data on planned gun purchase for 14 050 respondents (3612 with depressive symptoms).

^d^ Age is weighted by categories to reflect 12%, 34%, 33%, and 20% for 18 to 24 years, 24 to 44 years, 45 to 64 years, and 65 years or older, respectively.

^e^ No data on employment for 17 participants (4 with depressive symptoms).

^f^ Ideology is coded 1 = liberal to 7 = conservative; no data on ideology for 132 participants (37 with depressive symptoms).

## Results

Of 24 770 unique survey respondents, 6929 (28.0%) reported moderate or greater depressive symptoms; this group had a mean (SD) age of 38.18 (15.19) years; 4587 were female (66.2%), 406 were Asian (5.9%), 725 were Black (10.5%), 652 were Hispanic (6.8%), and 4902 were White (70.7%) (Table 1). Table 1 also includes reference values from the 2018 US Census American Community Survey, for comparison. Of those with depressive symptoms, 2167 (31.3%) reported current firearm ownership, and for 777 of these (35.9%), the first firearm purchase occurred during the COVID-19 pandemic. In logistic regression models, the presence of depressive symptoms was not significantly associated with firearm ownership (crude OR, 0.93; 95% CI, 0.86-1.00; adjusted OR, 1.07; 95% CI, 0.98-1.17; eTable 1 in the Supplement) but was associated with a greater likelihood of first-time firearm purchase during the pandemic (crude OR, 1.84; 95% CI, 1.62-2.08; adjusted OR, 1.77; 95% CI, 1.56-2.02; eTable 2 in the Supplement).

To validate survey-based estimates of firearm purchase and ownership results, we compared state-level results with administrative data, including an FBI database of background checks and a 2020 estimate of household firearm ownership rates. The proportion of survey respondents answering yes to household gun ownership was correlated with previously estimated firearm ownership by state (Pearson *r* = 0.90; *P* < .001; eFigure 1 in the Supplement). Similarly, the proportion of respondents answering yes to a recent purchase was correlated with FBI background checks per capita (Pearson *r* = 0.58; *P* < .001; eFigure 2 in the Supplement).

**Figure 1.**
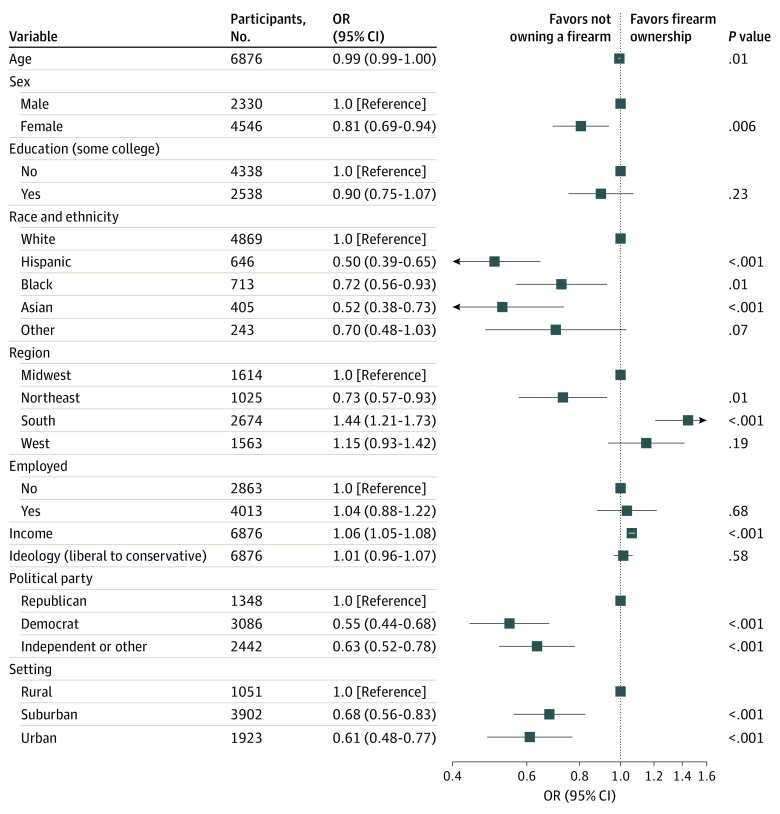
Sociodemographic Features Associated With Firearm Ownership Among Individuals With Moderate or Greater Depressive Symptoms OR indicates odds ratio.

We examined sociodemographic features associated with firearm ownership among individuals with depressive symptoms (Figure 1); the features significantly associated with the likelihood of ownership included younger age, male sex, White race, higher income, Republican party affiliation, residence in a rural area, and residence in the Southern United States. Restricting analyses to the 4270 individuals with moderate depression as well as suicidality, among whom 1364 (31.9%) reported gun ownership, yielded similar results (eFigure 3 in the Supplement).

We further characterized whether these risk factors differed from those among individuals without depression in logistic regression models by examining all survey respondents and including terms for interaction with depression (eTable 3 in the Supplement). Among the features showing main associations with depression, age and region of residence (Northeast vs Midwest) demonstrated statistically significant interactions with depression status. We observed opposing associations with age in respondents without depression (ie, greater risk among older participants; adjusted OR, 1.01; 95% CI, 1.00-1.01 among those without depression) and larger associations with region (adjusted OR, 0.47; 95% CI, 0.41-0.54 for Northeast vs Midwest among those without depression).

Table 2 lists the reasons for purchasing a firearm among 1860 first-time firearm purchasers, comparing those with and those without at least moderate depressive symptoms. For both groups, the most common reasons for a new purchase during the pandemic were protection against crime (1253 respondents [67.4%]), target shooting (751 respondents [40.4%]), and hunting (587 respondents [31.6%]). However, those with depressive symptoms were significantly less likely to report purchasing for protection (450 respondents [57.9%] vs 803 respondents [74.1%]) but more likely to report purchasing because of COVID-19 (129 respondents [16.6%] vs 84 respondents [7.8%]) and for protection against someone known to them (56 respondents [7.2%] vs 41 respondents [3.8%]).

**Table 2.** Reasons for Firearm Purchase During COVID-19 Pandemic Among Those Who Did Not Previously Own a Firearm

Reason(s) for purchase	Participants, No. (%)			*P* value
	Less than moderate depression (n = 1083)	Moderate or greater depression (n = 777)	Total (n = 1860)	
Hunting	330 (30.5)	257 (33.1)	587 (31.6)	.23
Target shooting	429 (39.6)	322 (41.4)	751 (40.4)	.43
Protection against crime	803 (74.1)	450 (57.9)	1253 (67.4)	<.001
Protection against government	199 (18.4)	141 (18.1)	340 (18.3)	.90
Because of COVID-19	84 (7.8)	129 (16.6)	213 (11.5)	<.001
Because of lockdown/restrictions	161 (14.9)	131 (16.9)	292 (15.7)	.24
Because of the election	184 (17.0)	107 (13.8)	291 (15.6)	.06
Protection against someone I know	41 (3.8)	56 (7.2)	97 (5.2)	.001

**Figure 2.**
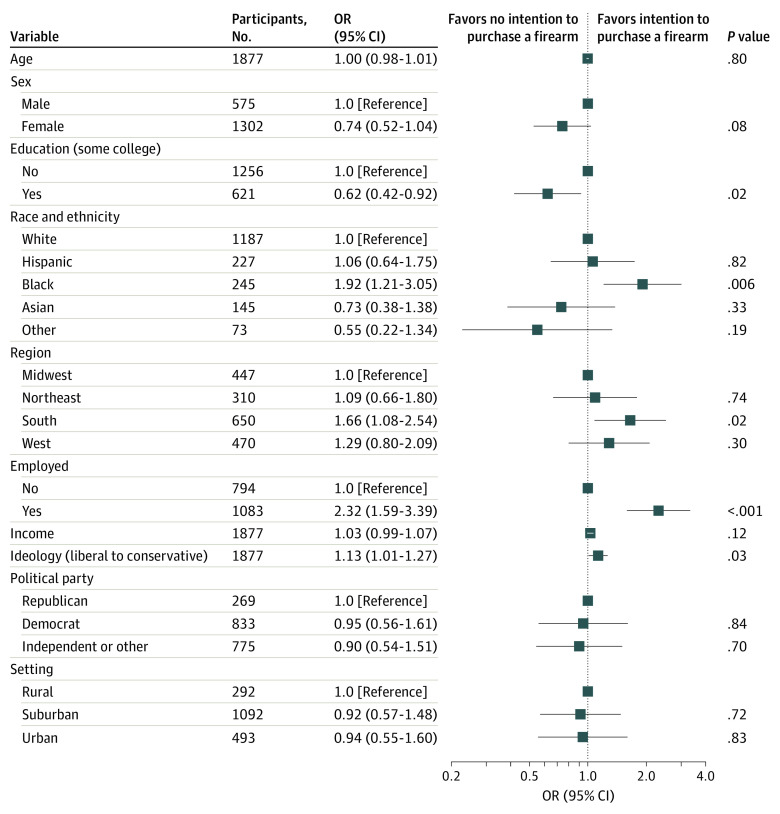
Sociodemographic Features Associated With Intention to Purchase a Firearm in the Near Future Among Individuals With Depressive Symptoms OR indicates odds ratio.

Next, we examined the intention to purchase a gun in the near future by individuals (n = 6232) who did not report current ownership. Current depressive symptoms were associated with a greater likelihood of considering a future firearm purchase (crude OR, 1.55; 95% CI, 1.27-1.89; adjusted OR, 1.53; 95% CI, 1.23-1.90; eTable 4 in the Supplement). Of the 1892 individuals with current depressive symptoms who do not currently own a firearm and were asked this question (Table 3), 330 (17.4%) with depressive symptoms responded that they were considering a firearm purchase in the near future; individuals with a lower educational level, those who are employed, and those identifying as more conservative in ideology were more likely to report an intention to purchase a firearm (Figure 2). Of note, many other features associated with current gun ownership, such as rural location and party affiliation, were not significantly associated with future purchase. Once again, restricting the cohort to 1153 individuals with both depression and suicidality but no current firearm, among whom 230 (19.9%) indicated an intention to purchase a firearm, yielded similar results (eFigure 4 in the Supplement).

**Table 3.** Sociodemographic Features of Survey Participants With or Without Moderate or Greater Symptoms of Major Depressive Disorder Who Do Not Currently Own a Firearm and Were Asked About Plans to Purchase a Firearm

Characteristic	Participants, No. (%)			*P* value
	Less than moderate depression (n = 4340)	Moderate or greater depressive symptoms (n = 1892)	Total (n = 6232)	
Planned gun purchase	523 (12.1)	330 (17.4)	853 (13.7)	<.001
Age, mean (SD), y	44.46 (17.08)	36.10 (13.90)	41.92 (16.63)	<.001
Sex				<.001
Female	2818 (64.9)	1315 (69.5)	4133 (66.3)	<.001
Male	1522 (35.1)	577 (30.5)	2099 (33.7)	NA
Race and ethnicity				
Asian	259 (6.0)	145 (7.7)	404 (6.5)	<.001
Black	420 (9.7)	231 (12.2)	651 (10.4)	
Hispanic	626 (14.4)	248 (13.1)	874 (14.0)	
White	2919 (67.3)	1195 (63.2)	4114 (66.0)	
Other	116 (2.7)	73 (3.9)	189 (3.0)	
Education (some college)	1963 (45.2)	624 (33.0)	2587 (41.5)	<.001
Currently employed^a^	2651 (61.1)	1094 (57.9)	3745 (60.1)	.02
Income, $10 000, mean (SD)	6.66 (5.03)	5.35 (4.89)	6.26 (5.02)	<.001
Region				
Midwest	1032 (23.8)	452 (23.9)	1484 (23.8)	.45
Northeast	783 (18.0)	312 (16.5)	1095 (17.6)	
South	1490 (34.3)	654 (34.6)	2144 (34.4)	
West	1035 (23.8)	474 (25.1)	1509 (24.2)	
Urbanicity				
Rural	605 (13.9)	295 (15.6)	900 (14.4)	.16
Suburban	2533 (58.4)	1102 (58.2)	3635 (58.3)	
Urban	1202 (27.7)	495 (26.2)	1697 (27.2)	
Political ideology, mean (SD)^b^	3.78 (1.57)	3.46 (1.55)	3.68 (1.57)	<.001
PHQ-9				
Suicidality	316 (7.3)	1153 (60.9)	1469 (23.6)	<.001
Suicidality score, mean (SD)	0.08 (0.31)	1.10 (1.08)	0.39 (0.80)	<.001
Mean score, mean (SD)	3.28 (2.98)	15.72 (4.66)	7.06 (6.75)	<.001

Abbreviations: NA, not applicable; PHQ-9, 9-item Patient Health Questionnaire.

^a^ No data on employment for 4 participants (1 with depressive symptoms).

^b^ Ideology is coded 1 = liberal to 7 = conservative; no data on ideology for 33 participants (11 with depressive symptoms).

## Discussion

In this study using survey data from 24 770 adults, we found that the rate of firearm ownership is common among individuals with moderate depressive symptoms, similar to the rates among those without such symptoms. These individuals were significantly more likely to have purchased a first firearm during the COVID-19 pandemic and more likely to report an intention to purchase a firearm in the near future than those who were not depressed. Individuals with depression who had purchased a firearm for the first time were less likely to have done so for protection against crime but more likely to say that they had purchased a firearm because of concern about COVID-19 or for protection against someone known to them. Otherwise, the sociodemographic features among those more likely to own firearms among respondents with depression were generally similar to those observed for respondents without depression.

A 2020 report established the substantial association between firearm ownership and suicide risk in the US; for suicides involving firearm, the hazard among men who own firearms increased nearly 8-fold, and the hazard among women increased 35-fold. These risks peaked after initial firearm acquisition.^4^ However, interaction with major depression is less frequently explored, with most prior work reflecting far smaller subpopulations. For example, in a study of 96 young adults with a lifetime history of suicidal ideation and either access to or interest in firearms, more than half reported firearm ownership.^9^ However, that study specifically selected participants with firearm interest, so it could not inform about the general population. Two prior studies^2,8^ specifically examined the association between depression, suicide, and firearm ownership. A study using data from the National Comorbidity Study and General Social Surveys nearly 3 decades ago found no association between depression and firearm ownership.^2^ A more recent study,^8^ reporting longitudinal data from 2004 to 2011, found an association between depression (as well as binge drinking and other substance use) with a greater likelihood of firearm ownership. Our finding that recent and planned purchases, but not current ownership, differed by depression status may help to explain these discordant findings; patterns of ownership change over time and may be influenced by perceived threats that also may affect mood state. The greater likelihood among individuals with depression of attributing a recent firearm purchase to fear of COVID-19 or fear of someone known to them is also consistent with a model of differential threat perception.

An important caveat in interpreting our results is the potential effect of the COVID-19 pandemic, which has been associated with marked increases in rates of depressive symptoms compared with historical baselines,^25,26^ although the extent of the increases remains subject to debate. The pandemic has also been associated with an increase in gun purchases.^5^ Our results provide some details about this increase; most gun purchases reflect concern about crime, followed by recreational use, but between 10% and 20% of respondents cite the need for protection from the government, concern about the US presidential election, or concern about COVID-19 or associated lockdown.

Despite the increase in gun purchases, to date, there does not appear to be an increase in suicides overall,^27^ although fatal overdoses have increased^28^ and young adults may represent an exception.^29^ As such, an urgent clinical question is whether the twin increases in depressive symptoms and gun ownership could be associated with a delayed increase in suicides. Certainly the presence of greater levels of suicidal ideation among individuals reporting a likelihood of purchasing a gun does not provide reassurance.

### Limitations

To maximize the representativeness of the US adult population as a whole, the survey methods adopt multiple strategies, including state-by-state quotas, followed by reweighting to reflect US census data. While many online surveys are subject to ascertainment bias because participants opt for topics of interest to them, this study did not use a firearm-specific survey, but rather this study used a generic opinion survey. Still, any survey-based design has limitations, and this study is no exception. In particular, probability samples have traditionally represented a criterion standard in surveys but would likely be 10 to 100 times more costly to collect. An AAPOR task force report notes the appropriateness of nonprobability samples for examining associations between variables, as in this study.^30^ A 2019 analysis found that an internet survey platform similar to that applied here yielded results similar to US national benchmarks,^14^ consistent with prior work showing that such surveys can yield representative samples.^15^ These findings have motivated large, well-resourced polling organizations to explore the use of nonprobability sampling.^13^ Moreover, it should be noted that the difficulty in reaching particular demographic subgroups, particularly for polling in recent election years, has raised questions about the ability of probability sampling to capture representative samples in the United States as well.^31^ Indeed, our validation analyses demonstrated a strong correlation between self-reported firearm ownership and purchasing and also demonstrated the best-available means of estimating these rates.

A final limitation of our survey is the lack of data on other characteristics that may influence the risk conferred by firearm ownership (eg, where and how firearms are stored) as well as other mental health comorbid conditions (such as substance use disorders). However, the goal of this analysis was to estimate the magnitude conferred by 2 major risk factors, rather than all reported risk factors. Validation of our survey-based approach to capturing firearm data suggests the feasibility of future studies incorporating such risk factors.

The incorporation of features capturing geographic region, political affiliation, and ideology may be criticized as limiting the generalizability of this work beyond the United States. In fairness, attitudes toward firearm possession in the United States, and access to firearms more generally, are difficult to extrapolate to other countries. The association of firearms with suicide (as well as homicide) in the United States suggests that, generalizable or not, it represents a crucial opportunity for intervention.^32^ Political beliefs may represent a key aspect of an individual’s identity and may explain the beliefs or behaviors that were not captured by typical sociodemographic features.

## Conclusions

Our results may facilitate more focused interventions to diminish suicide risk and increase firearm safety among individuals with 2 major suicide risk factors. While primary care screening for firearm ownership and safety has been widely advocated,^33^ such screening may pose particular challenges in relation to mental health. For example, a qualitative study of 37 individuals with depression identified a range of concerns related to firearm screening^34^—in particular, participant wariness about the response that admitting to firearm ownership might elicit. These challenges suggest that more narrowly directed or stratified strategies may be valuable, particularly in the context of depression or suicidality. In other words, knowing that the combination of gun ownership and suicidality is more common among particular groups of individuals may facilitate tailored messaging, outreach, and intervention.

## Notes

Supplement. **eTable 1.** Multiple Logistic Regression Model Examining Association of Presence or Absence of Moderate Depressive Symptoms and Sociodemographic Features With Current Firearm Ownership **eTable 2.** Multiple Logistic Regression Model Examining Association of Presence or Absence of Moderate Depressive Symptoms and Sociodemographic Features With Initial Firearm Purchase During the COVID-19 Pandemic **eTable 3.** Multiple Logistic Regression Model Examining Association of Presence or Absence of Moderate Depressive Symptoms and Sociodemographic Features With Initial Firearm Purchase During the COVID-19 Pandemic, Including Interactions With Major Depressive Disorder **eTable 4.** Multiple Logistic Regression Model Examining Association of Presence or Absence of Moderate Depressive Symptoms and Sociodemographic Features With Intention to Purchase a Firearm in the Near Future **eFigure 1.** Proportion by State of Households Owning Guns in 2016 Rand Data, Compared to Proportion of Respondents Who Report Owning Guns in April 2021 covidstates.org survey **eFigure 2.** Ratio by State of Firearm Background Checks to Adults 18 and Older, Compared to Proportion of Respondents Who Report Any Gun Purchase in 2 Years Prior to April 2021 covidstates.org survey **eFigure 3.** Sociodemographic Features Associated With Firearm Ownership Among Individuals With Moderate or Greater Depressive Symptoms And Suicidality **eFigure 4.** Sociodemographic Features Associated With Firearm Ownership Among Individuals With Moderate or Greater Depressive Symptoms and Suicidality
