# Supplementary material for: Prevalence of Firearm Ownership Among Individuals With Major Depressive Symptoms
Source: JAMA Netw Open. 2022 Mar 21;5(3):e223245. doi: 10.1001/jamanetworkopen.2022.3245 (PMC8938748; doi:10.1001/jamanetworkopen.2022.3245)
Supplement: Supplement. — eTable 1. Multiple Logistic Regression Model Examining Association of Presence or Absence of Moderate Depressive Symptoms and Sociodemographic Features With Current Firearm Ownership eTable 2. Multiple Logistic Regression Model Examining Association of Presence or Absence of Moderate Depressive Symptoms and Sociodemographic Features With Initial Firearm Purchase During the COVID-19 Pandemic eTable 3. Multiple Logistic Regression Model Examining Association of Presence or Absence of Moderate Depressive Symptoms and Sociodemographic Features With Initial Firearm Purchase During the COVID-19 Pandemic, Including Interactions With Major Depressive Disorder eTable 4. Multiple Logistic Regression Model Examining Association of Presence or Absence of Moderate Depressive Symptoms and Sociodemographic Features With Intention to Purchase a Firearm in the Near Future eFigure 1. Proportion by State of Households Owning Guns in 2016 Rand Data, Compared to Proportion of Respondents Who Report Owning Guns in April 2021 covidstates.org survey eFigure 2. Ratio by State of Firearm Background Checks to Adults 18 and Older, Compared to Proportion of Respondents Who Report Any Gun Purchase in 2 Years Prior to April 2021 covidstates.org survey eFigure 3. Sociodemographic Features Associated With Firearm Ownership Among Individuals With Moderate or Greater Depressive Symptoms And Suicidality eFigure 4. Sociodemographic Features Associated With Firearm Ownership Among Individuals With Moderate or Greater Depressive Symptoms and Suicidality [file jamanetwopen-e223245-s001.pdf]

Supplementary Online Content

Perlis RH, Simonson MD, Green J, et al. Prevalence of firearm ownership among individuals with major depressive symptoms. *JAMA Netw Open*. 2022;5(3):e223245. doi:10.1001/jamanetworkopen.2022.3245

eTable 1. Multiple Logistic Regression Model Examining Association of Presence or Absence of Moderate Depressive Symptoms and Sociodemographic Features With Current Firearm Ownership

eTable 2. Multiple Logistic Regression Model Examining Association of Presence or Absence of Moderate Depressive Symptoms and Sociodemographic Features With Initial Firearm Purchase During the COVID-19 Pandemic

eTable 3. Multiple Logistic Regression Model Examining Association of Presence or Absence of Moderate Depressive Symptoms and Sociodemographic Features With Initial Firearm Purchase During the COVID-19 Pandemic, Including Interactions With Major Depressive Disorder

eTable 4. Multiple Logistic Regression Model Examining Association of Presence or Absence of Moderate Depressive Symptoms and Sociodemographic Features With Intention to Purchase a Firearm in the Near Future

eFigure 1. Proportion by State of Households Owning Guns in 2016 Rand Data, Compared to Proportion of Respondents Who Report Owning Guns in April 2021 covidstates.org survey

eFigure 2. Ratio by State of Firearm Background Checks to Adults 18 and Older, Compared to Proportion of Respondents Who Report Any Gun Purchase in 2 Years Prior to April 2021 covidstates.org survey

eFigure 3. Sociodemographic Features Associated With Firearm Ownership Among Individuals With Moderate or Greater Depressive Symptoms and Suicidality

eFigure 4. Sociodemographic Features Associated With Firearm Ownership Among Individuals With Moderate or Greater Depressive Symptoms and Suicidality

This supplementary material has been provided by the authors to give readers additional information about their work.

© 2022 Perlis RH et al. *JAMA Network Open*.

Supplemental Materials

eTable 1. Multiple logistic regression model examining association of presence or absence of moderate depressive symptoms and sociodemographic features with current firearm ownership

------------------------------------	------------	---------	---------	---------
	OR	[95%	CI]	p-value
------------------------------------	-----------	--------	--------	--------
**Moderate depression**	1.0738	0.9851	1.1705	0.1054
Age (years)	1.0024	0.9999	1.0048	0.0559
Gender (female)	0.8518	0.7903	0.918	<.0001
Ethnicity (Hispanic)	0.6256	0.5424	0.7215	<.0001
Race (Black)	0.8646	0.7601	0.9834	0.0268
Race (Asian)	0.5174	0.4302	0.6222	<.0001
Race (Other)	0.8254	0.6455	1.0554	0.1261
Education (Some college or greater)	0.8495	0.7818	0.9229	0.0001
Employed	1.1158	1.0258	1.2137	0.0107
Income (per $10k)	1.0582	1.0499	1.0666	<.0001
Region (Northeast vs Midwest)	0.5246	0.4651	0.5918	<.0001
Region (South vs Midwest)	1.2243	1.1181	1.3406	<.0001
Region (West vs Midwest)	1.0897	0.9815	1.2097	0.1074
Suburban vs Rural	0.6907	0.6248	0.7636	<.0001
Urban vs Rural	0.5725	0.5081	0.6451	<.0001
Ideology	1.079	1.0491	1.1098	<.0001
Party (Democrat vs Republican)	0.5204	0.465	0.5823	<.0001
Party (Independent vs Republican)	0.6856	0.6209	0.757	<.0001
------------------------------------	------------	---------	---------	---------

© 2022 Perlis RH et al. *JAMA Network Open*.

eTable 2. Multiple logistic regression model examining association of presence or absence of moderate depressive symptoms and sociodemographic features with initial firearm purchase during the COVID-19 pandemic

------------------------------------	------------	---------	---------	---------
	OR	[95%	CI]	p-value
------------------------------------	-----------	--------	--------	--------
**Moderate depression**	1.7734	1.5547	2.0229	<.0001
Age (years)	0.9813	0.9772	0.9855	<.0001
Gender (female)	0.834	0.7333	0.9486	0.0057
Ethnicity (Hispanic)	0.7024	0.5511	0.8952	0.0043
Race (Black)	0.9527	0.7657	1.1854	0.6637
Race (Asian)	0.5367	0.384	0.75	0.0003
Race (Other)	1.1024	0.7731	1.572	0.5902
Education (Some college or greater)	0.9463	0.8146	1.0993	0.4703
Employed	1.479	1.2697	1.7229	<.0001
Income (per $10k)	1.0557	1.0426	1.069	<.0001
Region (Northeast vs Midwest)	0.6371	0.5132	0.791	<.0001
Region (South vs Midwest)	1.1964	1.0194	1.4042	0.0281
Region (West vs Midwest)	1.0049	0.8395	1.2028	0.9576
Suburban vs Rural	0.6718	0.5662	0.797	<.0001
Urban vs Rural	0.6546	0.5332	0.8036	0.0001
Ideology	1.05	0.9995	1.1031	0.0524
Party (Democrat vs Republican)	0.5317	0.4419	0.6398	<.0001
Party (Independent vs Republican)	0.5517	0.4638	0.6563	<.0001
------------------------------------	------------	---------	---------	---------

© 2022 Perlis RH et al. *JAMA Network Open*.

eTable 3. Multiple logistic regression model examining association of presence or absence of moderate depressive symptoms and sociodemographic features with initial firearm purchase during the COVID-19 pandemic, including interactions with major depressive disorder

--	------------	---------	---------	---------
	OR	[95% CI	]	p-value
--	-----------	--------	--------	--------
**Moderate depression**	2.4289	1.4329	4.1174	0.001
Age (years)	1.0052	1.0023	1.0081	0.0004
Gender (female)	0.8805	0.8079	0.9595	0.0037
Ethnicity (Hispanic)	0.6931	0.5827	0.8245	<.0001
Race (Black)	0.9321	0.8014	1.0842	0.3619
Race (Asian)	0.503	0.4037	0.6269	<.0001
Race (Other)	0.8827	0.6426	1.2125	0.4412
Education (Some college or greater)	0.844	0.7684	0.9269	0.0004
Employed	1.1656	1.0552	1.2877	0.0026
Income (per $10k)	1.0545	1.0451	1.0639	<.0001
Region (Northeast vs Midwest)	0.4696	0.4085	0.5397	<.0001
Region (South vs Midwest)	1.1553	1.0397	1.2838	0.0073
Region (West vs Midwest)	1.0777	0.9547	1.2167	0.2263
Suburban vs Rural	0.6927	0.6158	0.7792	<.0001
Urban vs Rural	0.5545	0.4822	0.6377	<.0001
Ideology	1.1153	1.0785	1.1533	<.0001
Party (Democrat vs Republican)	0.5173	0.4533	0.5903	<.0001
Party (Independent vs Republican)	0.7192	0.6422	0.8054	<.0001
**Interaction of moderate depression with…**				
Age (years)	0.9884	0.9826	0.9942	0.0001
Gender (female)	0.915	0.7669	1.0916	0.3237
Ethnicity (Hispanic)	0.7256	0.5351	0.9839	0.039
Race (Black)	0.7769	0.5806	1.0394	0.0892
Race (Asian)	1.0434	0.6986	1.5584	0.8357
Race (Other)	0.7958	0.4834	1.31	0.3691
Education (Some college or greater)	1.0643	0.8714	1.3	0.5413
Employed	0.8883	0.7341	1.0748	0.223
Income (per $10k)	1.0082	0.9896	1.0271	0.3898
Region (Northeast)	1.5553	1.1782	2.0532	0.0018
Region (South)	1.2506	1.0162	1.5391	0.0347
Region (West)	1.0675	0.8387	1.3586	0.5957
Suburban vs Rural	0.9793	0.7793	1.2306	0.8574
Urban vs Rural	1.0992	0.8379	1.4419	0.4946

© 2022 Perlis RH et al. *JAMA Network Open*.

Ideology	0.9098	0.8557	0.9673	0.0025
Party (Democrat vs Republican)	1.0549	0.8181	1.3603	0.6801
Party (Independent vs Republican)	0.8816	0.6968	1.1153	0.2935
--	------------	---------	---------	---------
* indicate interactions with p<0.05/17 (~0.003)				

© 2022 Perlis RH et al. *JAMA Network Open*.

eTable 4. Multiple logistic regression model examining association of presence or absence of moderate depressive symptoms and sociodemographic features with intention to purchase a firearm in the near future

------------------------------------	------------	---------	---------	---------
	OR	[95%	CI]	p-value
------------------------------------	-----------	--------	--------	--------
**Moderate depression**	1.5277	1.2278	1.9009	0.0001
Age (years)	0.9833	0.9766	0.9901	<.0001
Gender (female)	0.7115	0.5813	0.8709	0.001
Ethnicity (Hispanic)	0.9408	0.6866	1.2889	0.7039
Race (Black)	1.6191	1.2378	2.1178	0.0004
Race (Asian)	0.7009	0.4602	1.0677	0.098
Race (Other)	1.419	0.7838	2.5689	0.2479
Education (Some college or greater)	0.6822	0.5432	0.8568	0.001
Employed	1.7484	1.3811	2.2133	<.0001
Income (per $10k)	1.0194	0.997	1.0424	0.0905
Region (Northeast vs Midwest)	1.1384	0.8173	1.5855	0.4433
Region (South vs Midwest)	1.4897	1.1393	1.9478	0.0036
Region (West vs Midwest)	1.3317	0.9885	1.794	0.0597
Suburban vs Rural	0.8028	0.5961	1.0812	0.1482
Urban vs Rural	0.9262	0.6691	1.2821	0.6439
Ideology	1.1588	1.0777	1.246	0.0001
Party (Democrat vs Republican)	0.8088	0.5982	1.0935	0.1679
Party (Independent vs Republican)	0.7387	0.549	0.9939	0.0455
------------------------------------	------------	---------	---------	---------

© 2022 Perlis RH et al. *JAMA Network Open*.

Figures

eFigure 1.

Proportion by state of households owning guns in 2016 Rand data, compared to proportion of respondents who report owning guns in April 2021 covidstates.org survey

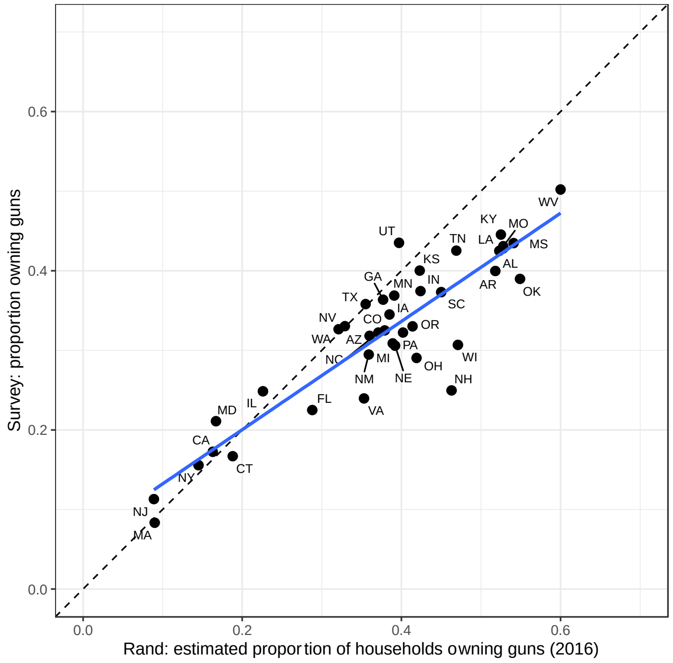

© 2022 Perlis RH et al. *JAMA Network Open*.

eFigure 2.

Ratio by state of firearm background checks to adults 18 and older, compared to proportion of respondents who report any gun purchase in 2 years prior to April 2021 covidstates.org survey

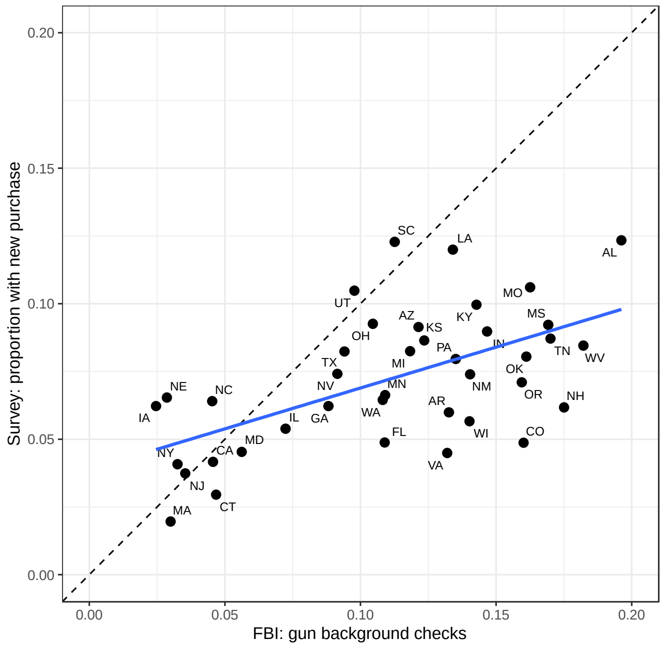

© 2022 Perlis RH et al. *JAMA Network Open*.

eFigure 3.

Sociodemographic features associated with firearm ownership among individuals with moderate or greater depressive symptoms and suicidality

Variable		N	Odds ratio		p
**Age**		4241		0.99 (0.99, 1.00)	0.076
**Gender**	Male	1763		Reference	
	Female	2478		0.77 (0.64, 0.94)	0.009
**Education (some college)**	FALSE	2545		Reference	
	TRUE	1696		0.80 (0.64, 1.02)	0.072
**Race**	White	2925		Reference	
	Hispanic	416		0.50 (0.37, 0.68)	<0.001
	Black	462		0.72 (0.53, 0.97)	0.033
	Asian	290		0.44 (0.30, 0.66)	<0.001
	Other	148		0.72 (0.44, 1.19)	0.202
**Region**	Midwest	938		Reference	
	Northeast	664		0.73 (0.54, 0.98)	0.037
	South	1637		1.48 (1.18, 1.85)	<0.001
	West	1002		1.04 (0.80, 1.35)	0.772
**Employed**	FALSE	1595		Reference	
	TRUE	2646		1.22 (0.99, 1.51)	0.066
**Income**		4241		1.05 (1.03, 1.07)	<0.001
**Ideology (liberal−>conservative)**		4241		0.97 (0.91, 1.03)	0.285
**Political party**	Republican	743		Reference	
	Democrat	2058		0.57 (0.43, 0.74)	<0.001
	Independent	1440		0.63 (0.48, 0.83)	<0.001
**Setting**	Rural	609		Reference	
	Suburban	2304		0.63 (0.49, 0.82)	<0.001
	Urban	1328		0.69 (0.52, 0.93)	0.013

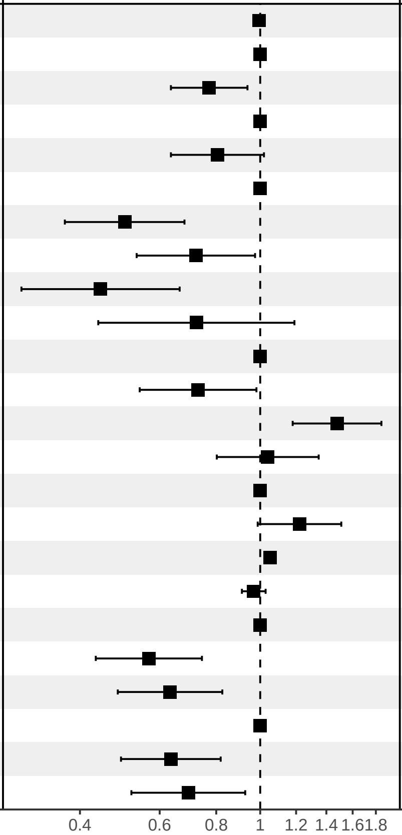

© 2022 Perlis RH et al. *JAMA Network Open*.

eFigure 4.

Sociodemographic features associated with firearm ownership among individuals with moderate or greater depressive symptoms and suicidality

Variable		N	Odds ratio		p
**Age**		1144		1.00 (0.99, 1.02)	0.601
**Gender**	Male	422		Reference	
	Female	722		0.86 (0.58, 1.28)	0.454
**Education (some college)**	FALSE	743		Reference	
	TRUE	401		0.73 (0.45, 1.19)	0.206
**Race**	White	712		Reference	
	Hispanic	136		1.05 (0.57, 1.94)	0.867
	Black	146		2.35 (1.33, 4.13)	0.003
	Asian	105		0.62 (0.29, 1.32)	0.215
	Other	45		0.70 (0.24, 1.97)	0.495
**Region**	Midwest	261		Reference	
	Northeast	188		1.19 (0.66, 2.15)	0.572
	South	389		1.80 (1.07, 3.05)	0.028
	West	306		1.30 (0.73, 2.34)	0.373
**Employed**	FALSE	467		Reference	
	TRUE	677		2.38 (1.50, 3.76)	<0.001
**Income**		1144		1.05 (1.00, 1.09)	0.031
**Ideology (liberal−>conservative)**		1144		1.07 (0.94, 1.21)	0.307
**Political party**	Republican	145		Reference	
	Democrat	538		0.74 (0.41, 1.35)	0.327
	Independent	461		0.74 (0.40, 1.38)	0.349
**Setting**	Rural	180		Reference	
	Suburban	629		1.00 (0.54, 1.85)	0.997
	Urban	335		1.18 (0.61, 2.29)	0.631

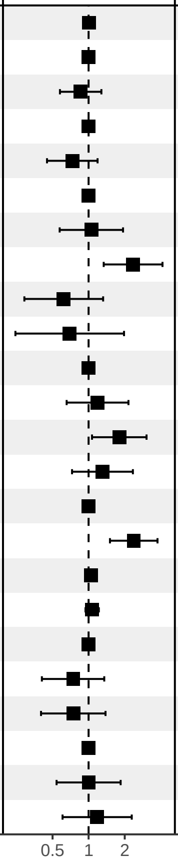

© 2022 Perlis RH et al. *JAMA Network Open*.
